# Research on an Optimized Quarter-Wavelength Resonator-Based Triboelectric Nanogenerator for Efficient Low-Frequency Acoustic Energy Harvesting

**DOI:** 10.3390/nano13101676

**Published:** 2023-05-19

**Authors:** Xiu Xiao, Ling Liu, Ziyue Xi, Hongyong Yu, Wenxiang Li, Qunyi Wang, Cong Zhao, Yue Huang, Minyi Xu

**Affiliations:** Dalian Key Lab of Marine Micro/Nano Energy and Self-Powered System, Marine Engineering College, Dalian Maritime University, Dalian 116026, China

**Keywords:** acoustic energy harvesting, low frequency, quarter-wavelength resonator, broadband

## Abstract

Sound wave is an extensively existing mechanical wave, especially in marine and industrial plants where low-frequency acoustic waves are ubiquitous. The effective collection and utilization of sound waves provide a fresh new approach to supply power for the distributed nodes of the rapidly developing Internet of Things technology. In this paper, a novel acoustic triboelectric nanogenerator (QWR-TENG) was proposed for efficient low-frequency acoustic energy harvesting. QWR-TENG consisted of a quarter-wavelength resonant tube, a uniformly perforated aluminum film, an FEP membrane, and a conductive carbon nanotube coating. Simulation and experimental studies showed that QWR-TENG has two resonance peaks in the low-frequency range, which effectively extends the response bandwidth of acoustic–electrical conversion. The structural optimized QWR-TENG has excellent electrical output performance, and the maximum output voltage, short-circuit current and transferred charge are 255 V, 67 μA, and 153 nC, respectively, under the acoustic frequency of 90 Hz and sound pressure level of 100 dB. On this basis, a conical energy concentrator was introduced to the entrance of the acoustic tube, and a composite quarter-wavelength resonator-based triboelectric nanogenerator (CQWR-TENG) was designed to further enhance the electrical output. Results showed that the maximum output power and the power density per unit pressure of CQWR-TENG reached 13.47 mW and 2.27 WPa^−1^m^−2^, respectively. Application demonstrations indicated that QWR/CQWR-TENG has good capacitor charging performance and is expected to realize power supply for distributed sensor nodes and other small electrical devices.

## 1. Introduction

With the rapid development of artificial intelligence (AI) and Internet of Things (IoT) technologies, the demand for distributed energy resources has increased dramatically [1,2,3,4,5]. As a clean, widespread, and sustainable energy source, sound waves are almost ubiquitous in the environment. In general, sound waves with frequencies ranging from 0 to 500 Hz are defined as low-frequency acoustic waves [6,7,8]. Such kinds of sound waves are characterized by low energy density and long wavelengths, and are able to penetrate obstacles and propagate over long distance. Notably, the acoustic waves in marine and industrial plants are mainly characterized by low-frequency and high sound pressure levels [9,10]. The application of acoustic energy collection technology is expected to provide a novel way to achieve acoustic–electrical conversion and supply power for the widely distributed sensors in the above-mentioned scenarios [11,12,13].

The basic principle of sound energy collection technology is to amplify sound waves using acoustic amplification devices such as Helmholtz resonator, tube resonator and acoustic metamaterials, and then convert the mechanical vibration energy into electrical output based on electromagnetic induction, piezoelectric effect, triboelectric effect or hybrid mechanism. In specific, acoustic energy harvesting devices based on electromagnetic induction use sound waves to drive conductors to cut magnetic lines in the magnetic field and generate induced current. Khan et al. [14] combined the Helmholtz resonator cavity with an electromagnetic generator for sound energy collection. The device generated a root-mean-square load voltage of 319.8 mV and a maximum power output of 1966.77 μW. On this basis, Izhar et al. [15] used a conical Helmholtz resonator to improve the electrical output of the sound energy collector. Results showed that the harvester had two resonance frequencies of 330.3 and 1332 Hz. At the first resonance frequency, the device generated a peak power of 177.2 mW under the incident sound pressure of 100 dB, which effectively improved the electrical output. However, due to the large scale of electromagnetic generator structures and the low conversion efficiency between sound waves and electromagnetic waves, external magnetic fields are usually required, resulting in low sound energy collection efficiency. Piezoelectric materials have good vibration sensitivity and can undergo mechanical deformation under the action of sound waves to generate electric fields. Yuan et al. [16] designed a sound energy collector consisting of an adjustable Helmholtz resonator, a fixed piezoelectric disk and a correction mass body. The device generated an output power of 3.49 μW and an energy conversion efficiency of 38.4% under an incident sound pressure of 100 dB. In addition to the traditional resonators, Qi et al. [17] proposed a new concept of using planar acoustic metamaterial to absorb and utilize high-frequency sound waves. Generally, the piezoelectric materials are susceptible to external interference and the output efficiency is relatively low. The triboelectric nanogenerator (TENG), driven by Maxwell displacement currents, can effectively convert distributed and disordered mechanical energy into electrical energy, and has shown great potential in energy collection [18,19,20,21,22] and self-powered systems [23,24,25,26,27,28,29]. In particular, the high vibration sensitivity of TENG makes it a possible high-efficient acoustic collection technology [20,30,31]. 

In recent years, researchers have conducted some studies on acoustic energy harvesting using TENG technology. In 2016, Yang et al. [32] designed a sound energy harvester by combining an adjustable Helmholtz resonant cavity with a contact-separation triboelectric nanogenerator. The design of a flexible film-based acoustic triboelectric nanogenerator was achieved for the first time, and good acoustic–electrical conversion efficiency was obtained at a resonant frequency of 240 Hz. Recently, Zhao et al. [33] proposed a dual-tube Helmholtz resonator-based triboelectric nanogenerator (HR-TENG). Compared to the previous acoustic TENGs based on traditional Helmholtz resonant cavities, the HR-TENG has a better output performance, with the maximum output voltage increased by 83%. The open-circuit voltage and short-circuit current reached 132 V and 32 µA at the optimal acoustic frequency. On this basis, Yuan et al. [34] presented an acoustic triboelectric nanogenerator using a conical Helmholtz resonator, which further improved the output performance of the sound energy harvester. Furthermore, Fan et al. [35] designed a paper-based triboelectric nanogenerator of 125 μm thickness to collect sound wave energy. The device generated a power density of 121 mW/m^2^ at the frequency of 320 Hz and a sound pressure level of 117 dB. Xu et al. [36] developed a self-powered laminated electrospun nanofiber triboelectric nanogenerator for sound energy harvesting. The open-circuit voltage was 170 V when two TENGs were stacked and operated at a frequency of 200 Hz. The above research shows that triboelectric nanogenerator technology has provided an effective way for efficient sound energy collection. However, the current researches mainly focus on acoustic waves with relatively high frequency, and can only achieve acoustic–electrical conversion in a narrow bandwidth, which greatly constrains the application of sound energy harvesters.

Quarter-wavelength resonator (QWR) is a typical acoustic amplifier with superior characteristics of simple structure, excellent sound pressure amplification effect, and wide resonance bandwidth, which has great potential for low-cost large-scale production [37,38,39]. Therefore, this paper presented a quarter-wavelength resonator-based triboelectric nanogenerator QWR-TENG for low-frequency acoustic energy harvesting. Based on the bimodal resonance characteristic of the acoustic–electrical coupling system in the low-frequency range, QWR-TENG has an excellent electrical output performance and effectively broadens the response bandwidth of acoustic TENG. Furthermore, a composite quarter-wavelength resonator-based triboelectric nanogenerator CQWR-TENG was proposed by introducing a conical energy concentrator to further improve the sound-electrical conversion efficiency and the electrical output. The as-designed sound energy harvester QWR/CQWR-TENG is expected to provide a low-power and cost-effective power solution for IoT technology.

## 2. Results and Discussion

### 2.1. Structure Design and Working Principle of QWR-TENG

**Figure 1:**
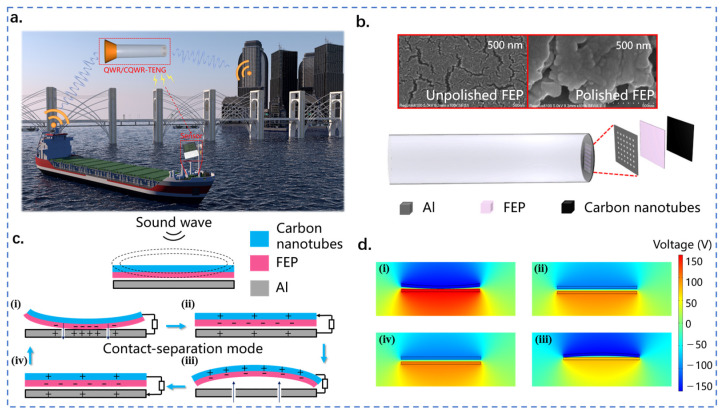
Structure and working principle of QWR-TENG. (**a**) Application scenario of QWR/CQWR-TENG in acoustic energy harvesting; (**b**) structure design of QWR-TENG; (**c**) working principle of QWR-TENG; (**d**) electrostatic field simulation by COMSOL Multiphysics.

Figure 1a depicts the application scenario of QWR/CQWR-TENG in various low-frequency sound sources such as ships and industrial plants. The QWR/CQWR-TENG can provide electrical energy for distributed wireless sensor nodes through acoustic energy harvesting. QWR-TENG consists of a quarter-wavelength resonant tube and a contact-separation TENG fixed on the closed side of the tube. As shown in Figure 1b, the TENG is composed of a uniformly perforated aluminum film, a flexible FEP film, and a carbon nanotube conductive ink layer. To improve the electrical output of QWR-TENG, the FEP film was sanded with 10,000 grit sandpaper. Figure 1b also shows the SEM images of the surface morphology of the FEP film before and after polishing. It can be seen that the surface roughness and the micro/nanostructures of the FEP film are significantly increased after sanding.

The sound waves produced by the sound source propagate in the form of vibration. In QWR-TENG, the quarter-wavelength resonant tube is used to amplify the acoustic waves, and then TENG technology is applied to convert the sound waves from vibration energy into electricity. The specific working principle is shown in Figure 1c. The FEP membrane is initially separated from the aluminum electrode, and the electrons in the aluminum film are free electrons. Under the excitation of sound waves, periodic pressure changes occur between the aluminum electrode and the FEP film, which cause the FEP membrane to vibrate and generate contact-separation with the aluminum film. When the FEP film comes into contact with the aluminum electrode, the FEP film becomes negatively charged due to its high electronegativity, and an equal amount of positive charge is generated on the aluminum electrode (Figure 1(ci)). Under the action of the changing sound pressure, the FEP membrane is separated from the aluminum electrode, and the positive and negative charges no longer overlap in the same plane, resulting in a dipole moment and potential difference between the surfaces. Therefore, free electrons are driven to flow between the top carbon nanotube electrode and the bottom aluminum electrode through an external circuit to balance the local electric field (Figure 1(cii)). The flow of electrons ceases when the separation between the two contact surfaces reaches its maximum (Figure 1(ciii)). Thereafter, the FEP film starts to move in the opposite direction and approach the bottom aluminum electrode. At this stage, the potential difference between the two electrodes weakens, and free electrons flow back to the top carbon nanotube electrode, thus generating a reverse current (Figure 1(civ)). Finally, the FEP film and the aluminum electrode come into contact again to complete a full power generation cycle (Figure 1(ci)). Figure 1d displays the potential changes on different electrodes obtained by COMSOL Multiphysics simulation. Apparently, the simulation results were consistent with the above analysis. Therefore, continuous alternative current (AC) pulses were generated in the external circuit of QWR-TENG and the conversion of mechanical energy into electrical energy was realized.

### 2.2. Bimodal Resonance Characteristic of QWR-TENG

**Figure 2:**
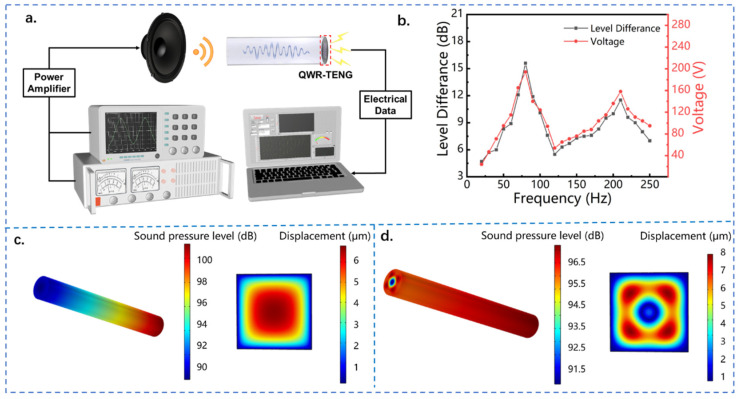
The bimodal resonance characteristic of QWR-TENG. (**a**) The diagram of the experimental system; (**b**) the sound pressure difference and open circuit voltage of QWR-TENG as a function of acoustic frequency; the acoustic field distribution in the quarter-wavelength resonant tube and the vibration mode of the FEP film (**c**) at the first-order resonant frequency and (**d**) at the second-order resonant frequency.

Figure 2a shows the schematic diagram of the experimental system for acoustic energy harvesting. The signal generator generates a sinusoidal electrical signal and drives the loudspeaker to produce sound waves. The frequency and sound pressure of the sound waves are controlled by the frequency and voltage of the electrical signal. During the experiment, the acoustic energy harvester is placed in an acrylic cover with soundproofing cotton on all sides to form a good sound insulation and shock absorption environment and ensure the accuracy of the experimental values. Under the excitation of sound waves, the triboelectric nanogenerator generates electrical signals through contact and separation between the dielectric material and the metal electrode. The electrical output is detected by an electrostatic high-impedance meter and collected by a data acquisition card.

The quarter-wavelength resonator is an important component of the acoustic energy harvester QWR-TENG, and its acoustic performance directly affects the electrical output of the device. QWR is a common acoustic amplification device, and its basic structure is a straight pipe with one end open and the other end closed. In order to systematically study the acoustic field of the quarter-wavelength resonator and its influence on the electrical output of QWR-TENG, the sound pressure difference of the quarter-wavelength tube and the output voltage of the triboelectric nanogenerator were measured at the inlet acoustic frequency of 30 to 250 Hz. As shown in Figure 2b, two peaks of sound pressure difference appeared in the resonator of QWR-TENG within the frequency band, which corresponded to the two natural resonance frequencies of 80 and 210 Hz, respectively. It is worth noting that the sound pressure difference caused by the first-order resonance in the quarter-wavelength resonant tube is significantly higher than that formed by the second-order resonance, that is, the sound pressure amplification factor attenuates at higher resonant mode. Specifically, the sound pressure difference reached 15.6 dB at the resonant frequency of *f* = 80 Hz, while the value was only 11.5 dB at the resonance frequency of *f* = 210 Hz. Consistent with the variation trend of the sound pressure difference, there were two output peaks in the open-circuit voltage of QWR-TENG as the frequency increased, which were 194 and 158 V, respectively. Since a larger acoustic pressure difference can enhance the contact-separation between the dielectric material and the aluminum electrode of TENG, a higher electrical output was generated at the first resonance frequency. According to the acoustic theory of pipelines, each resonance frequency corresponds to a specific vibration mode, and the resonant of the system is named the first-order characteristic mode and the second-order characteristic mode in order of increasing resonant frequency [40,41]. Due to the bimodal resonance characteristics of QWR, the QWR-TENG can be used to collect sound wave energy with a broad bandwidth in the low-frequency region.

Furthermore, the acoustic field distribution in the quarter-wavelength resonant tube and the vibration characteristic of the FEP film in the acoustic field were studied by using the acoustic-solid coupling numerical simulation method based on COMSOL 6.0 software. Figure 2c,d show the variation of sound pressure level (SPL) in the quarter-wavelength resonator and the vibration mode of the FEP membrane at the first-order resonance and the second-order resonance, respectively, in which the FEP film is subjected to a tensile stress of 10 N∙m^−1^. It can be seen that the quarter-wavelength resonator has a significant sound pressure amplification effect regardless of the resonant mode. Especially in the first-order resonance mode, the sound pressure level difference between the two ends of the resonant tube can reach 10 dB. In this resonant mode, the vibration displacement of the FEP membrane varies between 1 to 6 μm, and the displacement peak appears in the middle of the membrane and gradually decreases along the circumference. This indicates that both the highest positive pressure level and the lowest negative pressure level appear in the middle of the resonant cavity, which is beneficial for the contact-separation between the FEP membrane and the metal film and the electrical output of the acoustic TENG. When the inlet acoustic frequency exceeds the first resonant frequency, the acoustic field in the quarter-wavelength resonant tube gradually transforms to the second resonant mode. As demonstrated in Figure 2d, the sound pressure amplification effect decreases at the second-order resonance frequency, and the maximum sound pressure difference between the two ends of the resonator is about 5 dB. More importantly, the sound pressure in the resonator no longer exhibits a circular distribution, but presents multiple symmetrical distributions of sound pressure peaks. Correspondingly, the FEP membrane reveals multiple displacement peaks under the second resonant mode. For the proposed acoustic–electrical coupling system, the stress-strain behavior of the FEP film directly affects its contact-separation with the aluminum electrode, which in turn determines the electrical output of the device. According to the working principle of TENG [8], its output voltage follows:

(1) $$V_{oc}=\frac{\sigma x\left(t\right)}{\varepsilon_{0}}$$

where $V_{oc}\;$ is the open-circuit voltage, *σ* is the charge density, x(t) is the film displacement and $\varepsilon_{0}\;$ is the dielectric constant. It can be seen that when the material is determined, the dielectric constant is fixed, and the electrical output performance of TENG is related to the film displacement and the charge density of the surface. In the second-order resonance mode, although the peak displacement of the FEP film is enlarged, the specific vibration mode leads to a decrease in the effective contact area between the FEP membrane and the aluminum film, which in turn results in a reduction in the surface charge density. Therefore, the output voltage of QWR-TENG under the second-order resonance mode is reduced compared with that under the first-order resonance mode.

### 2.3. Output Performance of QWR-TENG

**Figure 3:**
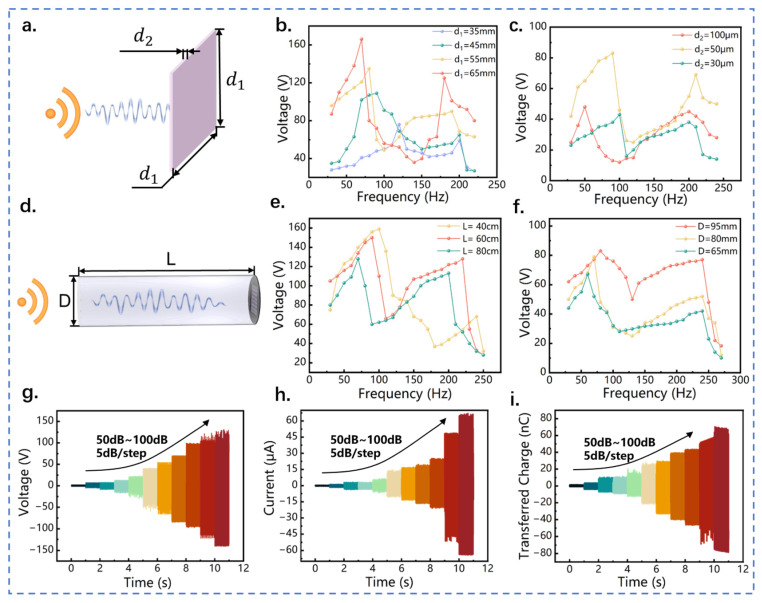
Effects of structural parameters and acoustic conditions on the electrical output of QWR-TENG. (**a**) Schematic diagram of the structural parameters of FEP membrane in the TENG power generation unit; effects of (**b**) power generation unit area and (**c**) FEP film thickness on the output voltage of QWR-TENG; (**d**) schematic diagram of the structural parameters of the quarter-wavelength resonator; effects of (**e**) the length and (**f**) diameter of the quarter-wavelength resonator on the output voltage of QWR-TENG; effects of incident pressure on the (**g**) open-circuit voltage, (**h**) short-circuit current; and (**i**) transferred charge of QWR-TENG.

In order to optimize the electrical output performance of QWR-TENG, sensitivity studies were conducted on the structural parameters of the acoustic–electrical coupling system, including the power generation unit area of TENG, the thickness of the FEP film, the length, and diameter of the quarter-wavelength resonant tube. The side length and thickness of the square FEP film are defined as *d*_1_ and *d*_2_, respectively, as shown in Figure 3a. Figure 3b displays the influence of the TENG power generation area on the electrical output performance of QWR-TENG. As the side length *d*_1_ of the FEP film increases from 35 to 65 mm, the first-order resonance frequency decreases from 120 to 70 Hz, but the change in second-order resonance frequency is small. This indicates that the response bandwidth of QWR-TENG increases as the effective power generation unit area is enlarged. In addition, the output voltage of QWR-TENG is positively correlated with the power generation unit area. As the power generation area increases, the deformation of the FEP film and its effective contact area with the aluminum electrode is increased. Accordingly, the peak voltage under the first-order and second-order resonance modes increases from 76 to 165 V and from 59 to 125 V, respectively. Similar variations in the output short-circuit current and transferred charge can also be found in Figure S1 of the Supplementary Materials. Therefore, for the acoustic triboelectric nanogenerator with a fixed quarter-wavelength resonant tube, the adoption of a larger TENG dielectric layer can not only increase the electrical output of the sound energy collector, but also extend the response bandwidth and operating range of the device.

The effect of the FEP film thickness *d*_2_ on the electrical output of QWR-TENG is shown in Figure 3c. Three types of FEP film with thicknesses of 30, 50, and 100 μm were tested and the effective area was set at 55 × 55 mm. The input acoustic wave frequency varied from 30 to 220 Hz with a step of 10 Hz during the experiment. On the one hand, as the thickness of the dielectric layer increased from 30 to 100 μm, the first-order resonance frequency decreased from 100 to 50 Hz, while the second-order resonance frequency remained basically unchanged. Therefore, increasing the thickness of the FEP film can expand the working bandwidth of QWR-TENG to a certain extent. Figure S2 in the supplementary reveals the short-circuit current and transferred charge. On the other hand, the electrical output of QWR-TENG did not change monotonically with the increase of film thickness, but showed a trend of first increasing and then decreasing. Specifically, under the first-order resonance mode, the output voltage of QWR-TENG reached 82 V when the FEP film thickness was set to 50 μm, which is 73% and 93% higher than that of the QWR-TENGs with a dielectric layer thickness of 30 and 100 μm, respectively. Furthermore, the peak output voltage was increased by 54% and 82%, respectively, at the second-order resonance frequency. The reason for this phenomenon is that when the thickness of the dielectric layer is increased (30 to 50 μm), its load impedance on the resonator enlarges gradually and is more suitable for the output impedance of the resonator, so the output voltage of QWR-TENG is increased. However, as the thickness of the FEP film is further enlarged (50 to 100 μm), the stiffness of the FEP film enhances significantly, which leads to a decrease in both the elastic deformation and effective contact area. As a result, the electrical output of QWR-TENG is decreased. Thus, in order to achieve the best output performance of QWR-TENG, it is necessary to select a dielectric layer material with a large area and an appropriate thickness for membrane-acoustic cavity coupling.

Transmission loss (TL) is the key indicator for acoustic performance evaluation [42]. Generally, it can be expressed as:

(2) $$TL=20lg\left|P_{i}/P_{t}\right|$$

where $P_{i}$ and $P_{t}$ represent the incident sound pressure and the transmitted sound pressure at the exit of the resonator, respectively. Therefore, a higher acoustic *TL* indicates a better resonance effect inside the resonant cavity. Acoustic studies show that the transmission loss *TL* of the quarter-wavelength resonator is related to quality factors, which mainly include the length, cross-sectional area, and impedance of the resonator. Therefore, the effects of the tube length and diameter of the quarter-wavelength resonator on the output performance of the QWR-TENG were studied. 

The length and diameter of the quarter-wavelength tube are defined as *L* and *D*, respectively, as shown in Figure 3d. Figure 3e shows the influence of quarter-wavelength tube length on the resonance frequency and output voltage of the QWR-TENG, in which the length is set as 40, 60, and 80 cm. It can be seen that the QWR-TENG has two output peaks regardless of the length, which corresponds to the two resonance frequencies of QWR. As the length of QWR decreased from 80 to 40 cm, the first-order resonance frequency increased from 70 to 100 Hz, but the output performance was enhanced. As shown in Figure S3 of the supplementary, similar variations can also be observed in short-circuit current and transferred charge. This indicates that the length of QWR-TENG can be adjusted to adapt to various acoustic frequencies. Since the present QWR-TENG is designed to harvest sound energy with low frequency, the QWR with a length of 60 cm was adopted for subsequent research by comprehensively considering the response frequency and electrical output performance.

The cross-sectional area of the quarter-wavelength tube is determined by its diameter, so the electrical output of three QWR-TENGs with different tube diameters was tested in the frequency range of 30 to 220 Hz. Figure 3f shows the output open-circuit voltage of the QWR-TENG, in which the diameter of the quarter-wavelength tube is 95, 80, and 65 mm, respectively. With the increase of pipe diameter and cross-sectional area, the first-order resonance frequency of QWR-TENG increases, but the second-order resonance frequency remains unchanged. More importantly, the electric output of QWR-TENG is effectively enhanced as the cross-sectional area increases. This is because a larger cross-sectional diameter indicates a higher impedance and a greater transmission loss of the resonator, thereby resulting in an increased electrical output of QWR-TENG. When the diameter of the quarter-wavelength resonant tube reached 95 mm, the peak open-circuit voltage of QWR-TENG reached 83 V, as shown in Figure 3f. Figure S4 in the supplementary reveals the short-circuit current and transferred charge, in which similar variation profiles can be observed. 

In addition, the impact of the inlet sound pressure level on the electric output of QWR-TENG is described in Figure 3g–i. The increase in inlet sound pressure amplifies the sound pressure difference at the outlet of the resonant tube, resulting in an enlarged deformation and radial displacement of the FEP membrane. Therefore, the electrical output of QWR-TENG increases with the incident sound pressure. More specifically, as the inlet sound pressure level raised from 50 to 100 dB, the open-circuit voltage, short-circuit current, and transferred charge of QWR-TENG increased from 4 to 255 V, from 1.2 to 67 μA, and from 2 to 153 nC, respectively. Figure S5 in the Supplementary Materials further demonstrates the electrical output of QWR-TENG under the incident sound pressure of 100 dB. 

### 2.4. Structure Design and Optimization of CQWR-TENG

The conical cavity structure can effectively guide the propagation of sound waves, and improve the sensitivity and effectiveness of sound radiation [43]. To further improve the output performance of the proposed acoustic–electrical coupling system, a conical energy concentrator was introduced to the open end of the quarter-wavelength tube to form a composite quarter-wavelength tube resonator-based triboelectric nanogenerator CQWR-TENG.

**Figure 4:**
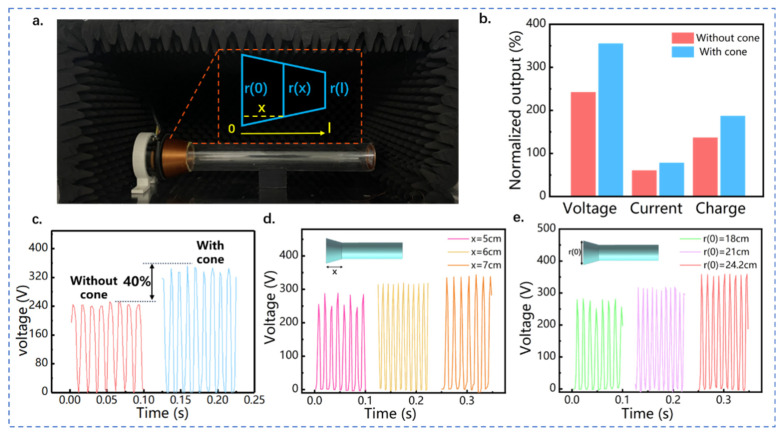
Structure design and output performance of CQWR-TENG. (**a**) Structure scheme of CQWR-TENG; (**b**) effect of conical cavity on the electrical output of the acoustic triboelectric nanogenerator; (**c**) comparison diagram of the output open-circuit voltage of QWR-TENG and CQWR-TENG; effects of (**d**) the length and (**e**) the open-end diameter of the conical cavity on the electrical output of CQWR-TENG.

The physical structure of CQWR-TENG and the simplified two-dimensional model of the conical cavity are shown in Figure 4a. Along the direction of sound wave propagation, the variation of the conical cavity diameter satisfies [44]:

(3) $$r\left(x\right)=r\left(l\right)-\frac{x}{l}\left[r\left(l\right)-r\left(0\right)\right]$$

where r(0) and r(l) are the inlet and outlet diameter of the conical cavity, respectively, l is the total length of the conical tube, and x is the length from the inlet end to the certain position inside the conical tube. According to the acoustic amplification theory of the conical cavity, the acoustic amplitude at the outlet of the conical cavity satisfies:

(4) $$A\left(x\right)=\frac{1}{r\left(x\right)}=\frac{l}{\left[lr\left(l\right)-x\left(r\left(l\right)-r\left(0\right)\right)\right]}$$

For a tapered conical cavity, r(x) decreases with the increase of x, so the amplitude of the sound wave  A(x) is magnified along the outlet direction of the conical tube. The sound pressure of acoustic waves is proportional to sound frequency and the square of the amplitude. Therefore, the increase in amplitude directly causes the amplification of sound pressure and sound intensity in the resonance cavity, thereby improving the electrical output of the acoustic energy collector. Figure 4b,c validate the enhancement effect of the conical concentrator on the output of the acoustic triboelectric nanogenerator. Under the same acoustic condition, the peak open-circuit voltage of QWR-TENG and CQWR-TENG are 242 and 348 V, respectively. Figure S6 in the supplementary displays the detailed output of QWR-TENG and CQWR-TENG under the acoustic frequency of 100 Hz and sound pressure level of 95.8 dB. Compared to QWR-TENG, the application of the conical concentrator increases the open-circuit voltage, short-circuit current, and transferred charge of the TENG by 43%, 33%, and 38%, respectively.

In addition, the structural parameters of the conical concentrator have a significant impact on the electrical output of TENG. The effect of the length of the conical cavity on the output performance of CQWR-TENG is displayed in Figure 4d, in which the lengths x are 5, 6, and 7 cm, respectively. Obviously, with the increase in the length of the conical concentrator, the output of CQWR-TENG is effectively enhanced, with the peak open-circuit voltage increasing from 285.2 to 339.1 V. The fact is that a larger length of the conical tube can make the reflection and interference phenomena more significant and enhance the accumulation of acoustic energy in the tube. This causes the enlargement of sound pressure and the increase of electrical output of CQWR-TENG. Figure 4e exhibits the effect of the opening diameter  r(0) of the conical concentrator on the output voltage. The open-circuit voltage of CQWR-TENG increased from 275.3 to 358 V as the opening diameter raised from 18 to 24.2 cm. This is because a larger opening diameter can diminish the reflection of sound waves at the entrance. In addition, the energy dissipation of sound waves along propagation can be reduced with a larger entrance diameter, thus maintaining a high amplification effect of sound pressure. In summary, increasing the opening diameter and the length of the conical energy concentrator is beneficial to the enhancement of sound pressure in CQWR-TENG. Therefore, the adoption of a conical energy concentrator is recommended within the available environmental space to increase the electrical output of acoustic TENG and improve the efficiency of acoustic–electrical energy conversion.

### 2.5. Demonstration of QWR/CQWR-TENG

**Figure 5:**
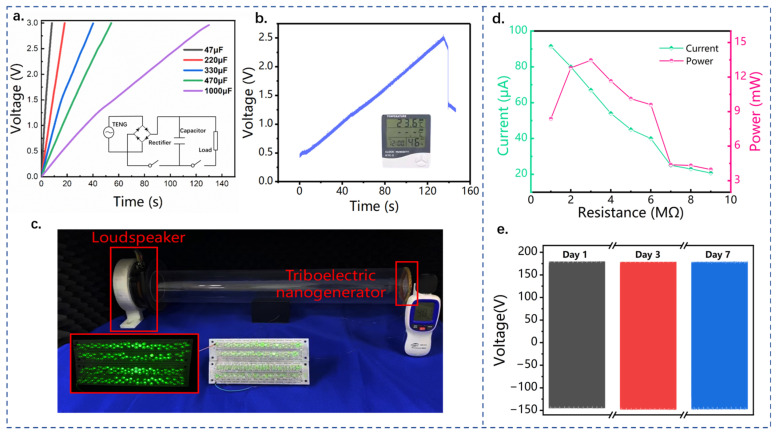
Power generation performance demonstration and output power test of QWR-TENG and CQWR-TENG. (**a**) Demonstration experiment of QWR-TENG charging capacitors with different capacities; (**b**) power supply experiment for temperature and humidity sensor; (**c**) schematic diagram of QWR-TENG simultaneously lighting 196 LEDs; (**d**) the variation profiles of the output current and power of CQWR-TENG with external load resistance; (**e**) durability demonstration of CQWR-TENG.

Demonstration experiments were carried out to verify the sound energy harvesting and power generation performance of QWR-TENG and CQWR-TENG, as shown in Figure 5. Figure 5a shows the circuit diagram and the results of QWR-TENG charging different capacitors. It takes about 9, 42, and 131 s for QWR-TENG to charge the capacitors of 47, 330, and 1000 μF to 3 V. This indicates that QWR-TENG has a good charging ability for capacitors and can supply electricity for low-power electronics. In addition, as exhibited in Figure 5b, after charging the 1000 μF capacitor to about 2.7 V, the humidity and temperature sensor was successfully lit by QWR-TENG. The Video S1 in the Supplementary Material shows that the sensor can operate continuously with the power supply of QWR-TENG. Figure 5c demonstrates the experiment of QWR-TENG lighting up 196 LEDs simultaneously under a sound pressure level of 90.1 dB and a frequency of 100 Hz, and the corresponding video can be found in Video S2 in the Supplementary Material. A demonstration experiment was also conducted in an onshore machinery compartment to test the sound energy harvesting ability of QWR-TENG. The experimental diagram can be found in Figure S6 in the supporting document, in which the sound waves were generated by the operation of a diesel engine. It can be seen that the QWR-TENG can output a voltage signal of 5~8 V at a certain distance from the sound source. The significant difference from the resonance frequency of QWR-TENG results in the limited electrical output of the device. In the future, the design of QWR-TENG structural parameters can be based on the actual industrial application scenarios to make its resonance frequency consistent with the frequency of the equipment, which is conducive for the application of QWR-TENG in sound energy harvesting.

The introduction of the conical accumulator can further enhance the sound energy collection and electrical output performance of the acoustic triboelectric nanogenerator. Figure 5d depicts the variation of CQWR-TENG output with external resistance under the acoustic condition of 90 Hz and 100 dB. As the external load resistance increased from 1 to 10 MΩ, the output current of CQWR-TENG gradually decreased from 91.5 to 20.7 μA. However, the output power of CQWR-TENG increased first and then decreased. Specifically, the maximum output power and the power density per unit pressure of CQWR-TENG reached 13.47 mW and 2.27 WPa^−1^m^−2^, respectively. The comparison with the earlier electromagnetic, piezoelectric, and triboelectric nanogenerator-based sound energy harvesters is presented in Table S1 of the supporting document [8,14,17,32,33,38,39,45,46,47,48], and the results indicate that the as-presented QWR-TENG has a superior performance in low-frequency acoustic–electrical conversion. Furthermore, the durability test result of CQWR-TENG is shown in Figure 5e. After seven days of operation, the electrical output decreased by about 1%, which confirmed the robustness of CQWR-TENG. Therefore, the as-designed QWR/CQWR-TENG has great application potential in the field of acoustic power generation, and is expected to be applied to specific acoustic scenarios for environmental low-frequency sound energy harvesting.

## 3. Experimental Section

### 3.1. Fabrication of QWR-TENG

The QWR-TENG consists of a quarter-wavelength resonator, an aluminum film with uniformly distributed sound holes, and an FEP film with an ink-printed electrode made of conductive carbon nanotubes. The quarter-wavelength resonator is made of a circular acrylic duct with a length of 60 cm, a diameter of 9.5 cm, and a thickness of 5 mm. For the fabrication of the triboelectric nanogenerator, FEP film was chosen as the electronegative material due to its high dielectric strength and good flexibility, and aluminum was selected as the electropositive material. The thickness of the FEP film is 50 μm, and one side of the film is treated with a corona discharge to facilitate electron transfer with the conductive carbon nanotube electrode. The aluminum film has a thickness of 0.1 mm and is uniformly perforated with one hundred small holes (diameter of 0.5 mm) for contact-separation of the TENG. The FEP membrane and the aluminum film are bonded together with adhesive tape, and then attached to a 3D-printed square ring to form the power generation unit of the TENG. Finally, the power generation unit was connected and fixed to the quarter-wavelength resonator using hot melt adhesive, forming the acoustic energy collector QWR-TENG.

### 3.2. Equipment of the Acoustic Energy Harvesting System

During the performance testing experiment, the QWR-TENG is installed in an acrylic cover with sound-absorbing cotton on all four walls, which has good sound insulation and shock absorption effects to ensure the accuracy of the experimental data. A loudspeaker, which is driven and adjusted by a function generator (YE1311) with sine waves, is used to produce sound waves. The sound is transmitted through a power amplifier (SA-5016) with an accuracy and resolution of 1.5 and 0.1 dB, respectively. The output signals including open-circuit voltage, short-circuit current, and transferred charge are measured by a Keithley 6514 electrostatic meter.

## 4. Conclusions

In this paper, a novel acoustic triboelectric nanogenerator QWR-TENG was firstly presented for the efficient collection of low-frequency sound wave energy. QWR-TENG was coupled by a quarter wavelength resonant tube and a contact-separation TENG to achieve acoustic–electrical conversion within a broad bandwidth. Simulation and experimental results showed that QWR-TENG was characterized by two resonance modes in the low-frequency range of 30~250 Hz, which can effectively extend the response bandwidth of the sound energy collector. The effects of structural parameters, including the length and cross-sectional diameter of the quarter-wavelength tube, the thickness of the FEP film, and the power generation area, were experimentally studied to enhance the electrical output performance of QWR-TENG. Results showed that the peak open-circuit voltage, short-circuit current, and transferred charge of the optimized QWR-TENG reached 255 V, 67 µA, and 153 nC, respectively. On this basis, a conical concentrator was introduced to the open end of the QWR to form a composite quarter-wavelength resonator-based triboelectric nanogenerator CQWR-TENG to further improve the electrical output of the acoustic collector. Experimental results showed that compared with QWR-TENG, the peak open-circuit voltage, short-circuit current, and transferred charge of CQWR-TENG increased by 43%, 33%, and 38%, respectively. More specifically, the maximum output power and the power density per unit pressure reached 13.47 mW and 2.27 WPa^−1^m^−2^. Demonstration experiments on capacitor charging and sensor power supply showed that the proposed QWR/CQWR-TENG has good output performance and the application potential in the field of acoustic energy collection. The response characteristics of being low-frequency and having a wide bandwidth make the QWR/CQWR-TENG promising for sound energy collection in marine and industrial plants, and to power distributed sensor nodes.

## Data Availability

Data are available on request from the authors.

## Supplementary Materials

The following supporting information can be downloaded at: https://www.mdpi.com/article/10.3390/nano13101676/s1, Figure S1: Effect of power generation unit area on (a) short-circuit current and (b) transferred charge of QWR-TENG; Figure S2: Effect of FEP film thickness on (a) short-circuit current and (b) transferred charge of QWR-TENG; Figure S3: Effect of the quarter-wavelength resonant tube length on (a) the short-circuit current and (b) transferred charge of QWR-TENG; Figure S4: Effect of the quarter-wavelength resonant tube diameter on (a) the short-circuit current and (b) transferred charge of QWR-TENG; Figure S5: Comparison diagram of the output performance of QWR-TENG and CQWR-TENG under the acoustic frequency of 100 Hz and sound pressure level of 95.8 dB. (a) Open-circuit voltage, (b) short circuit current and (c) transferred charge; Figure S6: Demonstration experiment of QWR-TENG collecting low-frequency sound energy in an onshore machinery compartment; Table S1: Acoustic energy harvesting capabilities of different acoustic harvesters; Video S1: The humidity and temperature sensor operates stably under the power supply of QWR-TENG; Video S2: The QWR-TENG powering 196 LEDs.
