# Supplementary material for: Research on an Optimized Quarter-Wavelength Resonator-Based Triboelectric Nanogenerator for Efficient Low-Frequency Acoustic Energy Harvesting
Source: Nanomaterials (Basel). 2023 May 19;13(10):1676. doi: 10.3390/nano13101676 (PMC10223044; doi:10.3390/nano13101676)
Supplement: Supplementary file 1 [file nanomaterials-13-01676-s001.zip › nanomaterials-2353462-supplementary/nanomaterials-2353462-supplementary.pdf]

nanomaterials

Research on an Optimized Quarter-Wavelength Resonator-Based Triboelectric Nanogenerator for Efficient Low-Frequency Acoustic Energy Harvesting

Xiu Xiao *, Ling Liu, Ziyue Xi, Hongyong Yu, Wenxiang Li, Qunyi Wang, Cong Zhao, Yue Huang and Minyi Xu *

Dalian Key Lab of Marine Micro/Nano Energy and Self-Powered System, Marine Engineering College, Dalian Maritime University, Dalian 116026, China
* Correspondence: xiaoxiu@dlmu.edu.cn (X.X.); xuminyi@dlmu.edu.cn (M.X.)

Supporting Figures

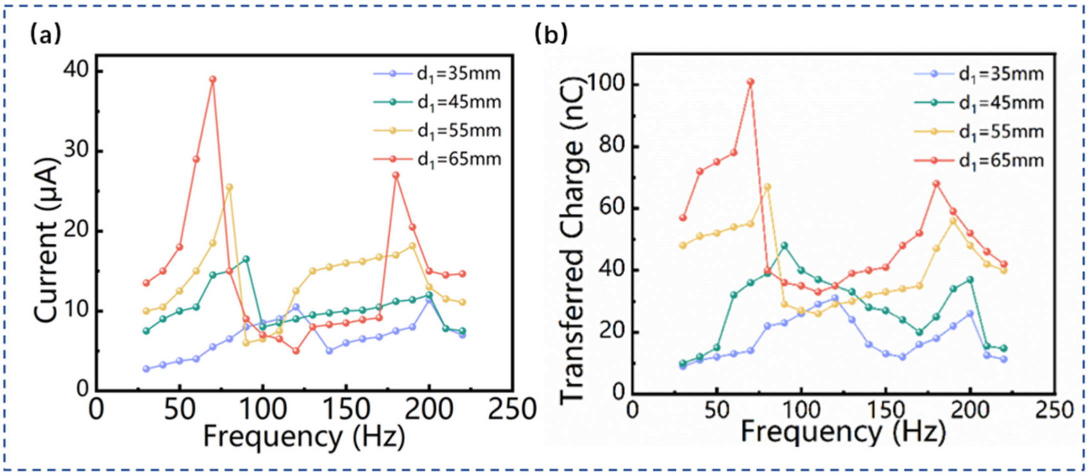

Figure S1. Effect of power generation unit area on (a) short-circuit current and (b) transferred charge of QWR-TENG.

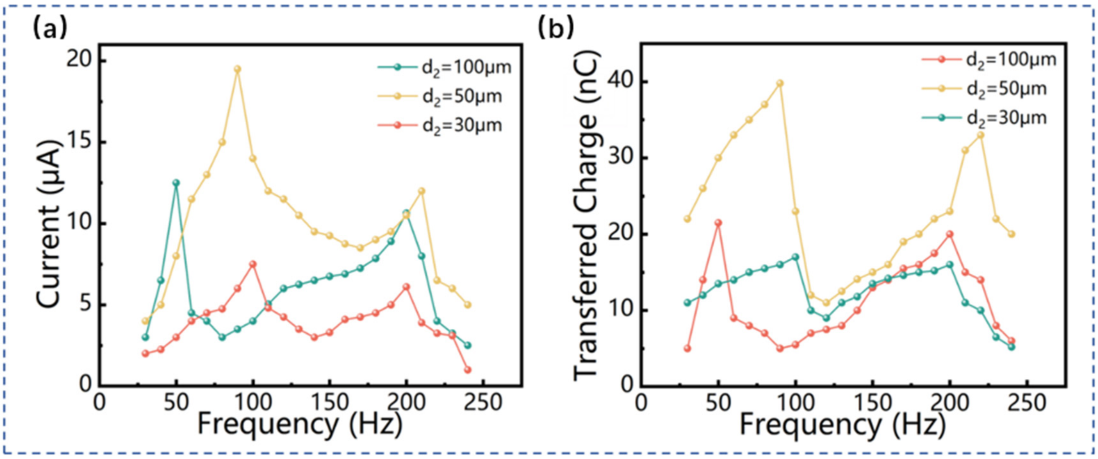

Figure S2. Effect of FEP film thickness on (a) short-circuit current and (b) transferred charge of QWR-TENG.

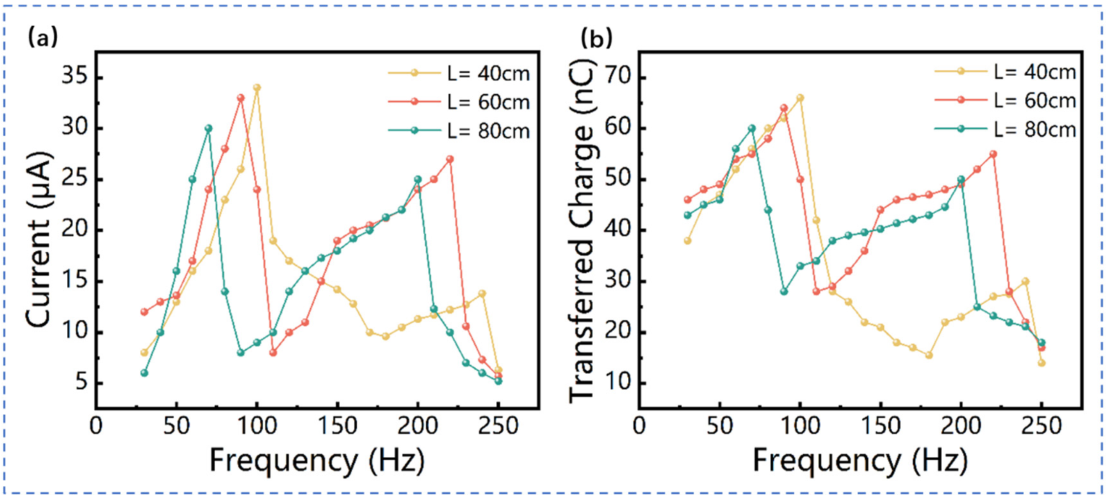

Figure S3. Effects of the quarter-wavelength resonant tube length on (a) the short-circuit current and (b) transferred charge of QWR-TENG.

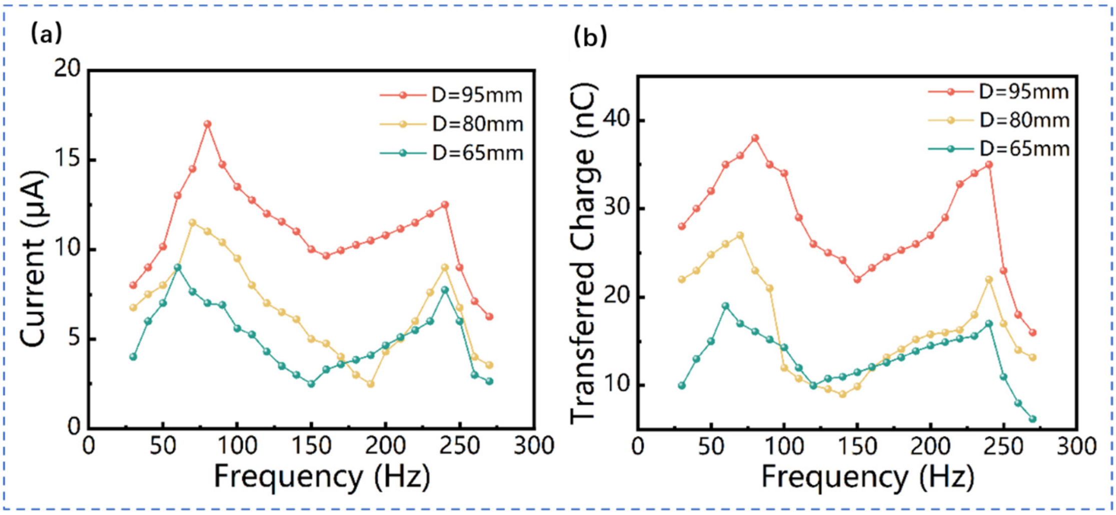

Figure S4. Effects of the quarter-wavelength resonant tube diameter on (a) the short-circuit current and (b) transferred charge of QWR-TENG.

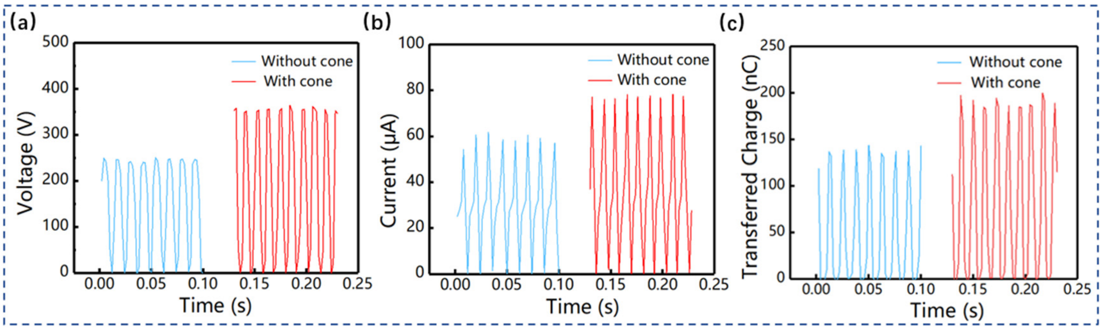

Figure S5. Comparison diagram of the output performances of QWR-TENG and CQWR-TENG under the acoustic frequency of 100 Hz and sound pressure level of 95.8 dB. (a) Open-circuit voltage, (b) short circuit current and (c) transferred charge.

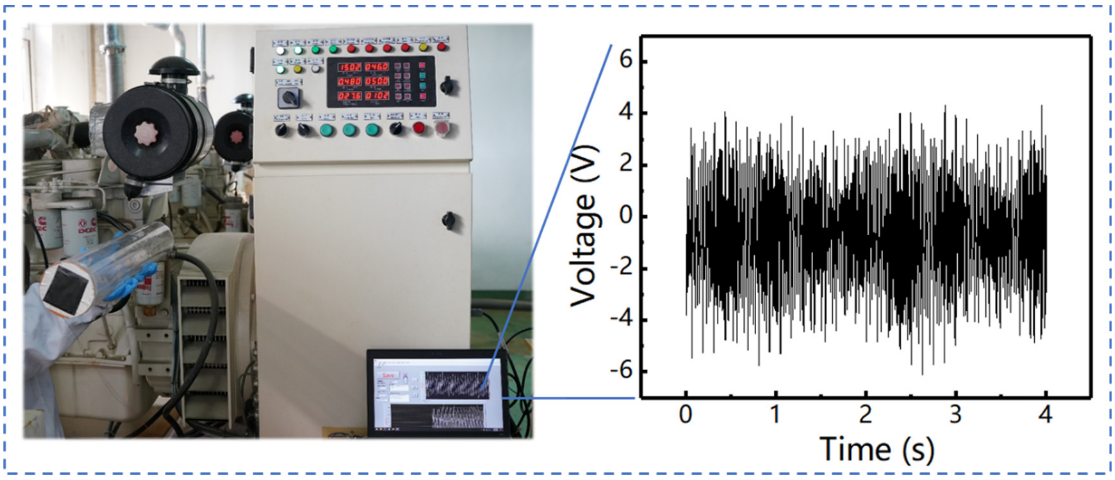

Figure S6. Demonstration experiment of QWR-TENG collecting low-frequency sound energy in an onshore machinery compartment.

Table S1. Acoustic energy harvesting capabilities of different acoustic harvesters

**Type**	**SPL (dB)**	**Frequency (Hz)**	**Power (μW)**	**Device dimensions (mm²)**	**Power density per unit (WPa⁻¹m⁻²)**	**Ref.**
Electromagnetic	125	143	2×10^3	589	0.094	[14]
	100	319	789.65	2.826×10^3	0.14	[48]
Piezoelectric	100	175	7.3	5×10^3	0.7×10^{-3}	[39]
	100	2257.5	8.8	3.6×10^3	1.22×10^{-3}	[17]
	100	100	0.3	100	1.5×10^{-3}	[45]
	110	199	650	800	0.13	[47]
TENG	106	100	670	4.9×10^3	0.02	[8]
	110	240	796	1.327×10^4	0.03	[33]
	100	/	4.33×10^3	5.02×10^3	0.43	[38]
	115	170	2.26×10^4	3.8×10^3	0.93	[46]
	89.1	80	2.1×10^3	2.03×10^3	1.82	[32]
Present	100	100	1.35×10^4	3.03×10^3	2.27	

Supporting Videos

The videos of QWR-TENG powering 196 LEDs and the humidity and temperature sensor can be found in Video S1 and Video S2.
